# Review of the families Mycteridae, Pythidae and Salpingidae (Coleoptera, Tenebrionoidea) of Bulgaria

**DOI:** 10.3897/BDJ.14.e185958

**Published:** 2026-03-09

**Authors:** Denis Gradinarov, Yana Petrova, Ognyan Sivilov

**Affiliations:** 1 Sofia University “St Kliment Ohridski”, Sofia, Bulgaria Sofia University “St Kliment Ohridski” Sofia Bulgaria; 2 National Genetic Laboratory, Sofia, Bulgaria National Genetic Laboratory Sofia Bulgaria

**Keywords:** Tenebrionoidea, *
Mycterus
*, *
Pytho
*, *
Salpingus
*, *
Vincenzellus
*, saproxylic beetles, distribution, Bulgaria

## Abstract

**Background:**

A total of seven beetle species from the families Mycteridae (two species), Pythidae (one species) and Salpingidae (four species) (Coleoptera, Tenebrionoidea) are known from Bulgaria. Beetles of this families are rarely recorded and three of the species are omitted in the last edition of the Catalogue of Palaearctic Coleoptera for the country, despite the presence of earlier reports.

**New information:**

In the present study, an updated checklist of the families Mycteridae, Pythidae and Salpingidae of Bulgaria is prepared. New data on the distribution of the species *Mycterus tibialis* Küster, 1850, *M. curculioides* (Fabricius, 1781) (Mycteridae), *Pytho depressus* (Linnaeus, 1767) (Pythidae), *Salpingus planirostris* (Fabricius, 1787), *S. ruficollis* (Linnaeus, 1760) and *Vincenzellus ruficollis* (Panzer, 1794) (Salpingidae) in the country are reported. Original photographs of all examined species are provided.

## Introduction

The small tenebrionoid families Mycteridae, Pythidae and Salpingidae (Coleoptera, Tenebrionoidea) include taxa distributed in both the Northern and Southern Hemispheres, with each family containing both temperate and at least one tropical genera (Pollock 2010a, Pollock 2010b, Lawrence et al. 2010). Historically, some genera of Mycteridae and Salpingidae have been classified within Pythidae (e.g. in Iablokoff-Khnzorian (1985)). Species of all three families are associated, at least during their larval development, with decaying wood of coniferous or deciduous species (Pollock 2010a, Pollock 2010b, Lawrence et al. 2010) and can, therefore, be considered saproxylic (Gimmel and Ferro 2018).

According to the second edition of the Catalogue of Palaearctic Coleoptera, four species of Mycteridae (Löbl et al. 2020), six species of Pythidae (Pollock and Iwan 2020) and 23 species of Salpingidae (Pollock et al. 2020) are known from Europe. The same edition lacks records of the families Mycteridae and Pythidae for Bulgaria, while, of the family Salpingidae, only three species are recorded: *Lissodema denticollis* (Gyllenhal, 1813), *Salpingus planirostris* (Fabricius, 1787) and *Vincenzellus ruficollis* (Panzer, 1794).

In the present work, we summarise all available literature data on the distribution of the species of the families Mycteridae, Pythidae and Salpingidae in Bulgaria. New data are provided for six of the species.

## Materials and methods

The material for this study was collected by the authors in the period 2017–2024 from different regions of Bulgaria. Adult beetles were collected by hand from flowering plants, net sweeping and incidental at light (for Mycteridae), hand collection under bark and in dead wood (for Pythidae and Salpingidae) and by flight interception trap (for Salpingidae). Flight interception traps were installed from spring to summer 2020 in well-preserved deciduous forests in Central Stara Planina Mountains and Rila Mountains and were examined monthly.

The photographs of the beetles' habitus and genital structures were taken with a Canon EOS R50 digital camera with Laowa 25 mm f/2.8 2.5-5X Ultra Macro or Laowa 90 mm f/2.8 2x Ultra Macro APO lens, mounted on a WeMacro rail (Wemacro, Shanghai, China).

The historical collection of Nikola Nedelkov, preserved in the Entomological collection of the National Museum of Natural History, Sofia, Bulgaria (NMNHS) was revised to clarify the species composition of the genus *Mycterus* Clairville, 1798 in Bulgaria. The newly-collected specimens are deposited in the Zoological Collection of Sofia University “St Kliment Ohridski”, Faculty of Biology, Sofia, Bulgaria (BFUS).

## Taxon treatments

### Mycterus (Eutryptes) tibialis

Küster, 1850

ED2A71F7-1D1B-5A82-BFF8-182225042B27

#### Materials

- **Type status:** Other material. **Occurrence:** recordedBy: N. Nedelkow; individualCount: 1; sex: female; previousIdentifications: *Mycterus umbellatarum* F.; occurrenceID: 714F3778-C738-5B7C-BE04-2C5834A7B950; **Location:** country: Bulgaria; locality: Burgas; **Identification:** identifiedBy: Denis Gradinarov; dateIdentified: 2025; **Record Level:** collectionCode: NMNHS
- **Type status:** Other material. **Occurrence:** recordedBy: N. Nedelkow; individualCount: 1; sex: female; occurrenceID: 83EA516B-55DA-5711-BBFF-6D5C4378D079; **Location:** country: Bulgaria; locality: Lyulin [Mts]; **Identification:** identifiedBy: Denis Gradinarov; dateIdentified: 2025; **Record Level:** collectionCode: NMNHS
- **Type status:** Other material. **Occurrence:** catalogNumber: BFUS-COL000574; recordedBy: Denis Gradinarov & Yana Petrova; individualCount: 1; sex: female; occurrenceID: 1A006AB5-E956-5923-9AE2-D9B9A8603AC8; **Location:** country: Bulgaria; stateProvince: Burgas; municipality: Primorsko; locality: Strandzha Mountains, 1.5 km SE of Novo Panicharevo Village; verbatimElevation: 133 m; decimalLatitude: 42.278800; decimalLongitude: 27.573583; geodeticDatum: WGS84; **Identification:** identifiedBy: Denis Gradinarov; dateIdentified: 2025; **Event:** samplingProtocol: net sweeping; verbatimEventDate: 07.vii.2017; habitat: meadows, edge of oak forest
- **Type status:** Other material. **Occurrence:** catalogNumber: BFUS-COL000575; recordedBy: Denis Gradinarov & Yana Petrova; individualCount: 1; sex: female; occurrenceID: C8440C3E-07CC-5417-816E-0B993E8441DB; **Location:** country: Bulgaria; stateProvince: Burgas; municipality: Primorsko; locality: Strandzha Mountains, 1.5 km SE of Novo Panicharevo Village; verbatimElevation: 133 m; decimalLatitude: 42.278800; decimalLongitude: 27.573583; geodeticDatum: WGS84; **Identification:** identifiedBy: Denis Gradinarov; dateIdentified: 2025; **Event:** samplingProtocol: net sweeping; verbatimEventDate: 07.vii.2017; habitat: meadows, edge of oak forest
- **Type status:** Other material. **Occurrence:** catalogNumber: BFUS-COL000576; recordedBy: Denis Gradinarov & Yana Petrova; individualCount: 1; sex: female; occurrenceID: A8C30BA6-CBF3-54A5-8EEB-3DAEB66DE3FC; **Location:** country: Bulgaria; stateProvince: Burgas; municipality: Primorsko; locality: Strandzha Mountains, 1.5 km SE of Novo Panicharevo Village; verbatimElevation: 133 m; decimalLatitude: 42.278800; decimalLongitude: 27.573583; geodeticDatum: WGS84; **Identification:** identifiedBy: Denis Gradinarov; dateIdentified: 2025; **Event:** samplingProtocol: net sweeping; verbatimEventDate: 07.vii.2017; habitat: meadows, edge of oak forest
- **Type status:** Other material. **Occurrence:** catalogNumber: BFUS-COL000577; recordedBy: Denis Gradinarov & Yana Petrova; individualCount: 1; sex: female; occurrenceID: FB5E6D24-07C1-5085-8CD7-ADA69A196706; **Location:** country: Bulgaria; stateProvince: Burgas; municipality: Primorsko; locality: Strandzha Mountains, 1.5 km SE of Novo Panicharevo Village; verbatimElevation: 133 m; decimalLatitude: 42.278800; decimalLongitude: 27.573583; geodeticDatum: WGS84; **Identification:** identifiedBy: Denis Gradinarov; dateIdentified: 2025; **Event:** samplingProtocol: net sweeping; verbatimEventDate: 07.vii.2017; habitat: meadows, edge of oak forest
- **Type status:** Other material. **Occurrence:** catalogNumber: BFUS-COL000578; recordedBy: Denis Gradinarov & Yana Petrova; individualCount: 1; sex: female; occurrenceID: 1AB0477F-F47B-5ACA-9C51-0840EB3F4B3C; **Location:** country: Bulgaria; stateProvince: Burgas; municipality: Primorsko; locality: Strandzha Mountains, 1.5 km SE of Novo Panicharevo Village; verbatimElevation: 133 m; decimalLatitude: 42.278800; decimalLongitude: 27.573583; geodeticDatum: WGS84; **Identification:** identifiedBy: Denis Gradinarov; dateIdentified: 2025; **Event:** samplingProtocol: net sweeping; verbatimEventDate: 07.vii.2017; habitat: meadows, edge of oak forest
- **Type status:** Other material. **Occurrence:** catalogNumber: BFUS-COL000579; recordedBy: Denis Gradinarov & Yana Petrova; individualCount: 1; sex: female; occurrenceID: 919EA524-3326-5AB9-AE01-773E0C7D3108; **Location:** country: Bulgaria; stateProvince: Burgas; municipality: Primorsko; locality: Strandzha Mountains, 1.5 km SE of Novo Panicharevo Village; verbatimElevation: 133 m; decimalLatitude: 42.278800; decimalLongitude: 27.573583; geodeticDatum: WGS84; **Identification:** identifiedBy: Denis Gradinarov; dateIdentified: 2025; **Event:** samplingProtocol: net sweeping; verbatimEventDate: 07.vii.2017; habitat: meadows, edge of oak forest
- **Type status:** Other material. **Occurrence:** catalogNumber: BFUS-COL000580; recordedBy: Denis Gradinarov & Yana Petrova; individualCount: 1; sex: female; occurrenceID: 43068BFC-6DE4-5206-B09B-3493C81E6ABB; **Location:** country: Bulgaria; stateProvince: Burgas; municipality: Primorsko; locality: Strandzha Mountains, 1.5 km SE of Novo Panicharevo Village; verbatimElevation: 133 m; decimalLatitude: 42.278800; decimalLongitude: 27.573583; geodeticDatum: WGS84; **Identification:** identifiedBy: Denis Gradinarov; dateIdentified: 2025; **Event:** samplingProtocol: net sweeping; verbatimEventDate: 07.vii.2017; habitat: meadows, edge of oak forest
- **Type status:** Other material. **Occurrence:** catalogNumber: BFUS-COL000581; recordedBy: Denis Gradinarov & Yana Petrova; individualCount: 1; sex: female; occurrenceID: 77408AC5-CA19-514D-A5B1-6D5EA2EFBDAE; **Location:** country: Bulgaria; stateProvince: Haskovo; municipality: Ivaylovgrad; locality: Eastern Rhodopes Mountains, NW of Plevun Village; verbatimElevation: 527 m; decimalLatitude: 41.498700; decimalLongitude: 25.980650; geodeticDatum: WGS84; **Identification:** identifiedBy: Denis Gradinarov; dateIdentified: 2025; **Event:** samplingProtocol: on flowers; verbatimEventDate: 06.vi.2020; habitat: meadows
- **Type status:** Other material. **Occurrence:** catalogNumber: BFUS-COL000582; recordedBy: Denis Gradinarov & Yana Petrova; individualCount: 1; sex: female; occurrenceID: CD8FD16B-2F57-58A3-AF4F-D29D9AA26996; **Location:** country: Bulgaria; stateProvince: Haskovo; municipality: Ivaylovgrad; locality: Eastern Rhodopes Mountains, NW of Plevun Village; verbatimElevation: 527 m; decimalLatitude: 41.498700; decimalLongitude: 25.980650; geodeticDatum: WGS84; **Identification:** identifiedBy: Denis Gradinarov; dateIdentified: 2025; **Event:** samplingProtocol: on flowers; verbatimEventDate: 06.vi.2020; habitat: meadows
- **Type status:** Other material. **Occurrence:** catalogNumber: BFUS-COL000583; recordedBy: Ognyan Sivilov; individualCount: 1; sex: male; occurrenceID: C67D0568-FCBB-5D53-99A3-D2C66841AE52; **Location:** country: Bulgaria; stateProvince: Veliko Tarnovo; municipality: Svishtov; locality: Middle Danubian Plain, NE of Hadzhidimitrovo Village; verbatimElevation: 72 m; decimalLatitude: 43.536650; decimalLongitude: 25.483567; geodeticDatum: WGS84; **Identification:** identifiedBy: Denis Gradinarov; dateIdentified: 2025; **Event:** samplingProtocol: net sweeping; verbatimEventDate: 20.v.2021; habitat: steppe vegetation
- **Type status:** Other material. **Occurrence:** catalogNumber: BFUS-COL000584; recordedBy: Ognyan Sivilov; individualCount: 1; sex: male; occurrenceID: 41C036A9-CD54-5A85-ACA1-F39AEA0A63EC; **Location:** country: Bulgaria; stateProvince: Veliko Tarnovo; municipality: Svishtov; locality: Middle Danubian Plain, NE of Hadzhidimitrovo Village; verbatimElevation: 72 m; decimalLatitude: 43.536650; decimalLongitude: 25.483567; geodeticDatum: WGS84; **Identification:** identifiedBy: Denis Gradinarov; dateIdentified: 2025; **Event:** samplingProtocol: net sweeping; verbatimEventDate: 20.v.2021; habitat: steppe vegetation
- **Type status:** Other material. **Occurrence:** catalogNumber: BFUS-COL000585; recordedBy: Ognyan Sivilov; individualCount: 1; sex: male; occurrenceID: AD972361-7139-5450-96F0-C964EBF83210; **Location:** country: Bulgaria; stateProvince: Veliko Tarnovo; municipality: Svishtov; locality: Middle Danubian Plain, NE of Hadzhidimitrovo Village; verbatimElevation: 72 m; decimalLatitude: 43.536650; decimalLongitude: 25.483567; geodeticDatum: WGS84; **Identification:** identifiedBy: Denis Gradinarov; dateIdentified: 2025; **Event:** samplingProtocol: net sweeping; verbatimEventDate: 20.v.2021; habitat: steppe vegetation
- **Type status:** Other material. **Occurrence:** catalogNumber: BFUS-COL000586; recordedBy: Ognyan Sivilov & Boyan Zlatkov; individualCount: 1; sex: male; occurrenceID: 4A561A70-3C2C-53E6-80E0-7F5448F91210; **Location:** country: Bulgaria; stateProvince: Veliko Tarnovo; municipality: Svishtov; locality: Middle Danubian Plain, NE of Hadzhidimitrovo Village; verbatimElevation: 81 m; decimalLatitude: 43.536483; decimalLongitude: 25.483017; geodeticDatum: WGS84; **Identification:** identifiedBy: Denis Gradinarov; dateIdentified: 2025; **Event:** samplingProtocol: at light; verbatimEventDate: 17.vi.2021; habitat: steppe vegetation
- **Type status:** Other material. **Occurrence:** catalogNumber: BFUS-COL000587; recordedBy: Ognyan Sivilov; individualCount: 1; sex: male; occurrenceID: 3DCAFF5C-EDE8-5875-998B-11FCEFFE0EDE; **Location:** country: Bulgaria; stateProvince: Dobrich; municipality: Kavarna; locality: Black Sea Coast, SW of Topola Village; verbatimElevation: 27 m; decimalLatitude: 43.409517; decimalLongitude: 28.261133; geodeticDatum: WGS84; **Identification:** identifiedBy: Denis Gradinarov; dateIdentified: 2025; **Event:** samplingProtocol: net sweeping; verbatimEventDate: 22.v.2021; habitat: steppe vegetation
- **Type status:** Other material. **Occurrence:** catalogNumber: BFUS-COL000588; recordedBy: Ognyan Sivilov; individualCount: 1; sex: male; occurrenceID: DEFAFBA6-43F5-525F-ACCA-0A2D32710589; **Location:** country: Bulgaria; stateProvince: Dobrich; municipality: Kavarna; locality: Black Sea Coast, SW of Topola Village; verbatimElevation: 27 m; decimalLatitude: 43.409517; decimalLongitude: 28.261133; geodeticDatum: WGS84; **Identification:** identifiedBy: Denis Gradinarov; dateIdentified: 2025; **Event:** samplingProtocol: net sweeping; verbatimEventDate: 22.v.2021; habitat: steppe vegetation
- **Type status:** Other material. **Occurrence:** catalogNumber: BFUS-COL000589; recordedBy: Ognyan Sivilov; individualCount: 1; sex: male; occurrenceID: B67950C4-A317-50A7-A59F-4D9879FF9BEB; **Location:** country: Bulgaria; stateProvince: Dobrich; municipality: Kavarna; locality: Black Sea Coast, SW of Topola Village; verbatimElevation: 27 m; decimalLatitude: 43.409517; decimalLongitude: 28.261133; geodeticDatum: WGS84; **Identification:** identifiedBy: Denis Gradinarov; dateIdentified: 2025; **Event:** samplingProtocol: net sweeping; verbatimEventDate: 22.v.2021; habitat: steppe vegetation
- **Type status:** Other material. **Occurrence:** catalogNumber: BFUS-COL000590; recordedBy: Denis Gradinarov; individualCount: 1; sex: male; occurrenceID: 24350425-1023-51AE-ADA9-079703B1F8CF; **Location:** country: Bulgaria; stateProvince: Blagoevgrad; municipality: Hadzhidimovo; locality: Falakro Mountains, NW of Beslen Village, road to Mesta River; verbatimElevation: 688 m; decimalLatitude: 41.478667; decimalLongitude: 23.962617; geodeticDatum: WGS84; **Identification:** identifiedBy: Denis Gradinarov; dateIdentified: 2021; **Event:** samplingProtocol: on *Tordylium* sp.; verbatimEventDate: 05.vi.2021; habitat: dirt road, xerothermic vegetation
- **Type status:** Other material. **Occurrence:** catalogNumber: BFUS-COL000591; recordedBy: Denis Gradinarov; individualCount: 1; sex: male; occurrenceID: 6796A968-9A74-56BB-889E-CA2C4B4E0F19; **Location:** country: Bulgaria; stateProvince: Blagoevgrad; municipality: Hadzhidimovo; locality: Falakro Mountains, NW of Beslen Village, road to Mesta River; verbatimElevation: 688 m; decimalLatitude: 41.478667; decimalLongitude: 23.962617; geodeticDatum: WGS84; **Identification:** identifiedBy: Denis Gradinarov; dateIdentified: 2021; **Event:** samplingProtocol: on *Tordylium* sp.; verbatimEventDate: 05.vi.2021; habitat: dirt road, xerothermic vegetation
- **Type status:** Other material. **Occurrence:** catalogNumber: BFUS-COL000592; recordedBy: Denis Gradinarov; individualCount: 1; sex: female; occurrenceID: 1DBCEAD9-B070-5BCF-AB24-270A3663D121; **Location:** country: Bulgaria; stateProvince: Blagoevgrad; municipality: Hadzhidimovo; locality: Falakro Mountains, NW of Beslen Village, road to Mesta River; verbatimElevation: 688 m; decimalLatitude: 41.478667; decimalLongitude: 23.962617; geodeticDatum: WGS84; **Identification:** identifiedBy: Denis Gradinarov; dateIdentified: 2021; **Event:** samplingProtocol: on *Tordylium* sp.; verbatimEventDate: 05.vi.2021; habitat: dirt road, xerothermic vegetation
- **Type status:** Other material. **Occurrence:** catalogNumber: BFUS-COL000593; recordedBy: Denis Gradinarov; individualCount: 1; sex: female; occurrenceID: DC2DF60F-1FFB-5588-91CA-5490478A1379; **Location:** country: Bulgaria; stateProvince: Blagoevgrad; municipality: Hadzhidimovo; locality: Falakro Mountains, NW of Beslen Village, road to Mesta River; verbatimElevation: 688 m; decimalLatitude: 41.478667; decimalLongitude: 23.962617; geodeticDatum: WGS84; **Identification:** identifiedBy: Denis Gradinarov; dateIdentified: 2021; **Event:** samplingProtocol: on *Tordylium* sp.; verbatimEventDate: 05.vi.2021; habitat: dirt road, xerothermic vegetation
- **Type status:** Other material. **Occurrence:** catalogNumber: BFUS-COL000594; recordedBy: Denis Gradinarov; individualCount: 1; sex: female; occurrenceID: 299242BF-E19B-5830-8C65-10EE9CCF301C; **Location:** country: Bulgaria; stateProvince: Blagoevgrad; municipality: Hadzhidimovo; locality: Falakro Mountains, NW of Beslen Village, road to Mesta River; verbatimElevation: 688 m; decimalLatitude: 41.478667; decimalLongitude: 23.962617; geodeticDatum: WGS84; **Identification:** identifiedBy: Denis Gradinarov; dateIdentified: 2021; **Event:** samplingProtocol: on *Tordylium* sp.; verbatimEventDate: 05.vi.2021; habitat: dirt road, xerothermic vegetation
- **Type status:** Other material. **Occurrence:** catalogNumber: BFUS-COL000595; recordedBy: Denis Gradinarov; individualCount: 1; sex: female; occurrenceID: 09D549C9-7B27-5F36-B405-A0B64F0E4F9B; **Location:** country: Bulgaria; stateProvince: Blagoevgrad; municipality: Hadzhidimovo; locality: Falakro Mountains, NW of Beslen Village; verbatimElevation: 692 m; decimalLatitude: 41.477900; decimalLongitude: 23.961967; geodeticDatum: WGS84; **Identification:** identifiedBy: Denis Gradinarov; dateIdentified: 2025; **Event:** samplingProtocol: on flowers; verbatimEventDate: 22.vi.2022; habitat: dirt road, edge of hornbeam forest
- **Type status:** Other material. **Occurrence:** catalogNumber: BFUS-COL000596; recordedBy: Denis Gradinarov; individualCount: 1; sex: female; occurrenceID: 20826DC2-99A1-5559-8554-3D40810AD7F0; **Location:** country: Bulgaria; stateProvince: Blagoevgrad; municipality: Hadzhidimovo; locality: Falakro Mountains, NW of Beslen Village; verbatimElevation: 692 m; decimalLatitude: 41.477900; decimalLongitude: 23.961967; geodeticDatum: WGS84; **Identification:** identifiedBy: Denis Gradinarov; dateIdentified: 2025; **Event:** samplingProtocol: on flowers; verbatimEventDate: 22.vi.2022; habitat: dirt road, edge of hornbeam forest
- **Type status:** Other material. **Occurrence:** catalogNumber: BFUS-COL000597; recordedBy: Denis Gradinarov; individualCount: 1; sex: female; occurrenceID: F425AD6A-EAAB-5CD1-811D-1744DB3E9227; **Location:** country: Bulgaria; stateProvince: Blagoevgrad; municipality: Hadzhidimovo; locality: Falakro Mountains, NW of Beslen Village; verbatimElevation: 692 m; decimalLatitude: 41.477900; decimalLongitude: 23.961967; geodeticDatum: WGS84; **Identification:** identifiedBy: Denis Gradinarov; dateIdentified: 2025; **Event:** samplingProtocol: on flowers; verbatimEventDate: 22.vi.2022; habitat: dirt road, edge of hornbeam forest
- **Type status:** Other material. **Occurrence:** catalogNumber: BFUS-COL000598; recordedBy: Denis Gradinarov; individualCount: 1; sex: female; occurrenceID: 3D08A245-B969-5610-8686-EC8481411726; **Location:** country: Bulgaria; stateProvince: Blagoevgrad; municipality: Hadzhidimovo; locality: Falakro Mountains, NW of Beslen Village; verbatimElevation: 692 m; decimalLatitude: 41.477900; decimalLongitude: 23.961967; geodeticDatum: WGS84; **Identification:** identifiedBy: Denis Gradinarov; dateIdentified: 2025; **Event:** samplingProtocol: on flowers; verbatimEventDate: 22.vi.2022; habitat: dirt road, edge of hornbeam forest
- **Type status:** Other material. **Occurrence:** catalogNumber: BFUS-COL000599; recordedBy: Ognyan Sivilov; individualCount: 1; sex: male; occurrenceID: F05DC106-65C8-5D89-AB44-A8799667B8AE; **Location:** country: Bulgaria; stateProvince: Montana; municipality: Lom; locality: Western Danubian Plain, W of Orsoya Village; verbatimElevation: 35 m; decimalLatitude: 43.777600; decimalLongitude: 23.079467; geodeticDatum: WGS84; **Identification:** identifiedBy: Denis Gradinarov; dateIdentified: 2025; **Event:** samplingProtocol: net sweeping; verbatimEventDate: 07.vi.2021; habitat: riverine meadows
- **Type status:** Other material. **Occurrence:** catalogNumber: BFUS-COL000600; recordedBy: Ognyan Sivilov; individualCount: 1; sex: male; occurrenceID: B5352CCE-2296-55C3-97CA-9744CAF32EFC; **Location:** country: Bulgaria; stateProvince: Dobrich; municipality: Kavarna; locality: Eastern Danubian Plain, South Dobrudzha, NE of Vidno Village; verbatimElevation: 76 m; decimalLatitude: 43.581683; decimalLongitude: 28.413633; geodeticDatum: WGS84; **Identification:** identifiedBy: Denis Gradinarov; dateIdentified: 2023; **Event:** samplingProtocol: net sweeping; verbatimEventDate: 08.vi.2021; habitat: steppe vegetation
- **Type status:** Other material. **Occurrence:** catalogNumber: BFUS-COL000601; recordedBy: Ognyan Sivilov; individualCount: 1; sex: male; occurrenceID: F4A709F5-5CD4-535E-BA9E-EF608FE206D3; **Location:** country: Bulgaria; stateProvince: Dobrich; municipality: Kavarna; locality: Eastern Danubian Plain, South Dobrudzha, NE of Vidno Village; verbatimElevation: 76 m; decimalLatitude: 43.581683; decimalLongitude: 28.413633; geodeticDatum: WGS84; **Identification:** identifiedBy: Denis Gradinarov; dateIdentified: 2025; **Event:** samplingProtocol: net sweeping; verbatimEventDate: 08.vi.2021; habitat: steppe vegetation
- **Type status:** Other material. **Occurrence:** catalogNumber: BFUS-COL000602; recordedBy: Ognyan Sivilov; individualCount: 1; sex: male; occurrenceID: D650138A-12F3-54DB-86DB-9A433A94890E; **Location:** country: Bulgaria; stateProvince: Dobrich; municipality: Kavarna; locality: Eastern Danubian Plain, South Dobrudzha, NE of Vidno Village; verbatimElevation: 76 m; decimalLatitude: 43.581683; decimalLongitude: 28.413633; geodeticDatum: WGS84; **Identification:** identifiedBy: Denis Gradinarov; dateIdentified: 2025; **Event:** samplingProtocol: net sweeping; verbatimEventDate: 08.vi.2021; habitat: steppe vegetation
- **Type status:** Other material. **Occurrence:** catalogNumber: BFUS-COL000603; recordedBy: Ognyan Sivilov; individualCount: 1; sex: male; occurrenceID: 6E6C2904-0BE1-52E6-86A9-C78C5D48BD1D; **Location:** country: Bulgaria; stateProvince: Dobrich; municipality: Kavarna; locality: Eastern Danubian Plain, South Dobrudzha, NE of Vidno Village; verbatimElevation: 76 m; decimalLatitude: 43.581683; decimalLongitude: 28.413633; geodeticDatum: WGS84; **Identification:** identifiedBy: Denis Gradinarov; dateIdentified: 2025; **Event:** samplingProtocol: net sweeping; verbatimEventDate: 08.vi.2021; habitat: steppe vegetation
- **Type status:** Other material. **Occurrence:** catalogNumber: BFUS-COL000604; recordedBy: Ognyan Sivilov; individualCount: 1; sex: male; occurrenceID: 17A56E04-32CA-53B1-8AF0-C96194FC1940; **Location:** country: Bulgaria; stateProvince: Dobrich; municipality: Kavarna; locality: Eastern Danubian Plain, South Dobrudzha, NE of Vidno Village; verbatimElevation: 76 m; decimalLatitude: 43.581683; decimalLongitude: 28.413633; geodeticDatum: WGS84; **Identification:** identifiedBy: Denis Gradinarov; dateIdentified: 2025; **Event:** samplingProtocol: net sweeping; verbatimEventDate: 08.vi.2021; habitat: steppe vegetation
- **Type status:** Other material. **Occurrence:** catalogNumber: BFUS-COL000605; recordedBy: Ognyan Sivilov; individualCount: 1; sex: female; occurrenceID: 1239BA35-53FF-5B39-A016-0EC0B988DB0B; **Location:** country: Bulgaria; stateProvince: Dobrich; municipality: Kavarna; locality: Eastern Danubian Plain, South Dobrudzha, NE of Vidno Village; verbatimElevation: 76 m; decimalLatitude: 43.581683; decimalLongitude: 28.413633; geodeticDatum: WGS84; **Identification:** identifiedBy: Denis Gradinarov; dateIdentified: 2025; **Event:** samplingProtocol: net sweeping; verbatimEventDate: 08.vi.2021; habitat: steppe vegetation
- **Type status:** Other material. **Occurrence:** catalogNumber: BFUS-COL000606; recordedBy: Denis Gradinarov & Yana Petrova; individualCount: 1; sex: male; occurrenceID: 5E4BDD01-94A0-55AA-A8AE-09A76706F4F9; **Location:** country: Bulgaria; stateProvince: Yambol; municipality: Elhovo; locality: Derventski Vazvisheniya Hills, E of Malko Kirilovo Village; verbatimElevation: 292 m; decimalLatitude: 42.031733; decimalLongitude: 26.629400; geodeticDatum: WGS84; **Identification:** identifiedBy: Denis Gradinarov; dateIdentified: 2025; **Event:** samplingProtocol: net sweeping; verbatimEventDate: 28.vi.2022; habitat: roadside meadows, edge of oak forest
- **Type status:** Other material. **Occurrence:** catalogNumber: BFUS-COL000607; recordedBy: Denis Gradinarov & Yana Petrova; individualCount: 1; sex: female; occurrenceID: D6148E1C-3DA4-5D38-8A10-ED6E8A37D152; **Location:** country: Bulgaria; stateProvince: Yambol; municipality: Elhovo; locality: Derventski Vazvisheniya Hills, E of Malko Kirilovo Village; verbatimElevation: 292 m; decimalLatitude: 42.031733; decimalLongitude: 26.629400; geodeticDatum: WGS84; **Identification:** identifiedBy: Denis Gradinarov; dateIdentified: 2025; **Event:** samplingProtocol: net sweeping; verbatimEventDate: 28.vi.2022; habitat: roadside meadows, edge of oak forest
- **Type status:** Other material. **Occurrence:** catalogNumber: BFUS-COL000608; recordedBy: Denis Gradinarov & Yana Petrova; individualCount: 1; sex: female; occurrenceID: FE9A72AC-8B43-58D2-837C-62200E7B635A; **Location:** country: Bulgaria; stateProvince: Yambol; municipality: Elhovo; locality: Derventski Vazvisheniya Hills, E of Malko Kirilovo Village; verbatimElevation: 292 m; decimalLatitude: 42.031733; decimalLongitude: 26.629400; geodeticDatum: WGS84; **Identification:** identifiedBy: Denis Gradinarov; dateIdentified: 2025; **Event:** samplingProtocol: net sweeping; verbatimEventDate: 28.vi.2022; habitat: roadside meadows, edge of oak forest
- **Type status:** Other material. **Occurrence:** catalogNumber: BFUS-COL000609; recordedBy: Denis Gradinarov & Yana Petrova; individualCount: 1; sex: female; occurrenceID: 8BD3DA4D-3ED7-5BF1-85C6-284F6DC81E9D; **Location:** country: Bulgaria; stateProvince: Yambol; municipality: Elhovo; locality: Derventski Vazvisheniya Hills, E of Malko Kirilovo Village; verbatimElevation: 292 m; decimalLatitude: 42.031733; decimalLongitude: 26.629400; geodeticDatum: WGS84; **Identification:** identifiedBy: Denis Gradinarov; dateIdentified: 2025; **Event:** samplingProtocol: net sweeping; verbatimEventDate: 28.vi.2022; habitat: roadside meadows, edge of oak forest
- **Type status:** Other material. **Occurrence:** catalogNumber: BFUS-COL000610; recordedBy: Denis Gradinarov & Yana Petrova; individualCount: 1; sex: female; occurrenceID: 09EA7444-26B7-5B58-B38B-5D6B6767D84A; **Location:** country: Bulgaria; stateProvince: Haskovo; municipality: Lyubimets; locality: Sakar Mountains, N of Yerusalimovo Village; verbatimElevation: 174 m; decimalLatitude: 41.905050; decimalLongitude: 26.101233; geodeticDatum: WGS84; **Identification:** identifiedBy: Denis Gradinarov; dateIdentified: 2025; **Event:** samplingProtocol: on flowers; verbatimEventDate: 26.v.2023; habitat: roadsive vegetation
- **Type status:** Other material. **Occurrence:** catalogNumber: BFUS-COL000611; recordedBy: Denis Gradinarov & Yana Petrova; individualCount: 1; sex: female; occurrenceID: 6BE3CA0E-7B92-587B-9454-9ADAFD0DE896; **Location:** country: Bulgaria; stateProvince: Haskovo; municipality: Lyubimets; locality: Sakar Mountains, N of Yerusalimovo Village; verbatimElevation: 174 m; decimalLatitude: 41.905050; decimalLongitude: 26.101233; geodeticDatum: WGS84; **Identification:** identifiedBy: Denis Gradinarov; dateIdentified: 2025; **Event:** samplingProtocol: on flowers; verbatimEventDate: 26.v.2023; habitat: roadsive vegetation
- **Type status:** Other material. **Occurrence:** catalogNumber: BFUS-COL000612; recordedBy: Denis Gradinarov & Yana Petrova; individualCount: 1; sex: male; occurrenceID: 2051B6F5-542E-5111-B7E1-2960082CA667; **Location:** country: Bulgaria; stateProvince: Haskovo; municipality: Lyubimets; locality: Sakar Mountains, SW of Yerusalimovo Village; verbatimElevation: 117 m; decimalLatitude: 41.892367; decimalLongitude: 26.094967; geodeticDatum: WGS84; **Identification:** identifiedBy: Denis Gradinarov; dateIdentified: 2025; **Event:** samplingProtocol: net sweeping; verbatimEventDate: 11.v.2024; habitat: roadside vegetation, pasture
- **Type status:** Other material. **Occurrence:** catalogNumber: BFUS-COL000613; recordedBy: Denis Gradinarov & Yana Petrova; individualCount: 1; sex: female; occurrenceID: 517B5E83-C832-5956-8BE9-099987B61AF5; **Location:** country: Bulgaria; stateProvince: Haskovo; municipality: Lyubimets; locality: Sakar Mountains, SW of Yerusalimovo Village; verbatimElevation: 117 m; decimalLatitude: 41.892367; decimalLongitude: 26.094967; geodeticDatum: WGS84; **Identification:** identifiedBy: Denis Gradinarov; dateIdentified: 2025; **Event:** samplingProtocol: net sweeping; verbatimEventDate: 11.v.2024; habitat: roadside vegetation, pasture
- **Type status:** Other material. **Occurrence:** catalogNumber: BFUS-COL000614; recordedBy: Denis Gradinarov & Yana Petrova; individualCount: 1; sex: female; occurrenceID: E1A632B7-7519-5CA9-92D8-5E4E5D55899B; **Location:** country: Bulgaria; stateProvince: Haskovo; municipality: Lyubimets; locality: Sakar Mountains, SW of Yerusalimovo Village; verbatimElevation: 117 m; decimalLatitude: 41.892367; decimalLongitude: 26.094967; geodeticDatum: WGS84; **Identification:** identifiedBy: Denis Gradinarov; dateIdentified: 2025; **Event:** samplingProtocol: net sweeping; verbatimEventDate: 11.v.2024; habitat: roadside vegetation, pasture

#### Description

**Figure 1.**
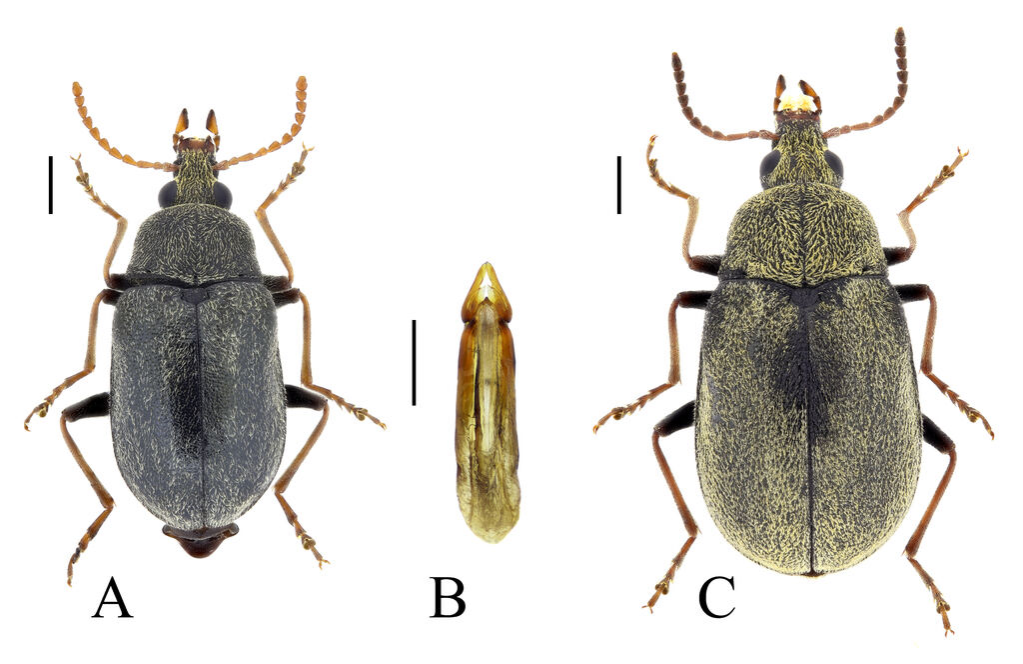
Habitus of *Mycterus tibialis* Küster, 1850 from Falakro Mountains, Bulgaria: **A** male (BFUS-COL000590); **B** aedeagus of the same specimen; **C** female (BFUS-COL000592). Scale bars: 1 mm (A, C); 0.5 mm (B).

The species is characterised, in particular, by its relatively short rostrum and by at least partially light legs and antennae (Fig. 1). The aedeagus is more elongate compared to that of *M. curculioides*, with narrow parameres (Iablokoff-Khnzorian 1985) (Fig. 1). It can be distinguished from the closely-related Mediterranean species *M. umbellatarum* (Fabricius, 1787), in particular by the parallel sides of the rostrum (narrowed apically in *M. umbellatarum*) and by the position of the antennae, attached at a greater distance from the eyes (Iablokoff-Khnzorian 1985).

### Mycterus (Mycterus) curculioides

(Fabricius, 1781)

B3847237-F67B-5EFD-8CB0-D4CF1F3212DA

#### Materials

- **Type status:** Other material. **Occurrence:** catalogNumber: BFUS-COL000615; recordedBy: Denis Gradinarov; individualCount: 1; sex: female; occurrenceID: F4ACF54C-81BE-56C6-B1B2-88D6764AAF98; **Location:** country: Bulgaria; stateProvince: Blagoevgrad; municipality: Razlog; locality: Rila Mountains, W of Razlog, Predel Рass; verbatimElevation: 1149 m; decimalLatitude: 41.884500; decimalLongitude: 23.347967; geodeticDatum: WGS84; **Identification:** identifiedBy: Denis Gradinarov; dateIdentified: 2025; **Event:** samplingProtocol: on *Crataegus monogyna*; verbatimEventDate: 06.vi.2021; habitat: meadows and shrubs
- **Type status:** Other material. **Occurrence:** catalogNumber: BFUS-COL000616; recordedBy: Denis Gradinarov; individualCount: 1; sex: female; occurrenceID: 72076D96-1282-58AF-8032-C94CC7F49A9B; **Location:** country: Bulgaria; stateProvince: Blagoevgrad; municipality: Razlog; locality: Rila Mountains, W of Razlog, Predel Рass; verbatimElevation: 1149 m; decimalLatitude: 41.884500; decimalLongitude: 23.347967; geodeticDatum: WGS84; **Identification:** identifiedBy: Denis Gradinarov; dateIdentified: 2025; **Event:** samplingProtocol: on *Crataegus monogyna*; verbatimEventDate: 06.vi.2021; habitat: meadows and shrubs
- **Type status:** Other material. **Occurrence:** catalogNumber: BFUS-COL000617; recordedBy: Denis Gradinarov; individualCount: 1; sex: male; occurrenceID: B0CD1F87-B7A4-51BB-8086-9202B5280AF1; **Location:** country: Bulgaria; stateProvince: Haskovo; municipality: Harmanli; locality: Sakar Mountains, NW of Rogozinovo Village, left bank of Maritsa River; verbatimElevation: 75 m; decimalLatitude: 41.926917; decimalLongitude: 25.934967; geodeticDatum: WGS84; **Identification:** identifiedBy: Denis Gradinarov; dateIdentified: 2025; **Event:** samplingProtocol: on Apiaceae; verbatimEventDate: 20.vii.2021; habitat: riverine vegetation
- **Type status:** Other material. **Occurrence:** catalogNumber: BFUS-COL000618; recordedBy: Denis Gradinarov; individualCount: 1; sex: male; occurrenceID: A7D684BE-3FF1-5B3D-89CC-7C7849A6BC57; **Location:** country: Bulgaria; stateProvince: Haskovo; municipality: Harmanli; locality: Sakar Mountains, NW of Rogozinovo Village, left bank of Maritsa River; verbatimElevation: 75 m; decimalLatitude: 41.926917; decimalLongitude: 25.934967; geodeticDatum: WGS84; **Identification:** identifiedBy: Denis Gradinarov; dateIdentified: 2025; **Event:** samplingProtocol: on Apiaceae; verbatimEventDate: 20.vii.2021; habitat: riverine vegetation
- **Type status:** Other material. **Occurrence:** catalogNumber: BFUS-COL000619; recordedBy: Denis Gradinarov; individualCount: 1; sex: female; occurrenceID: D9F0A439-197B-5B04-A87C-61907AD26841; **Location:** country: Bulgaria; stateProvince: Haskovo; municipality: Topolovgrad; locality: Sakar Mountains, SE of Ustrem Village, the road to Radovets Village; verbatimElevation: 203 m; decimalLatitude: 41.976050; decimalLongitude: 26.483767; geodeticDatum: WGS84; **Identification:** identifiedBy: Denis Gradinarov; dateIdentified: 2025; **Event:** samplingProtocol: on flowers; verbatimEventDate: 23.vii.2021; habitat: meadows, edge of oak forest
- **Type status:** Other material. **Occurrence:** catalogNumber: BFUS-COL000620; recordedBy: Yana Petrova; individualCount: 1; sex: female; occurrenceID: 8192A706-3D1B-5C45-8B07-F7DBE9A659FF; **Location:** country: Bulgaria; stateProvince: Haskovo; municipality: Topolovgrad; locality: Sakar Mountains, SE of Ustrem Village, the road to Radovets Village; verbatimElevation: 203 m; decimalLatitude: 41.976050; decimalLongitude: 26.483767; geodeticDatum: WGS84; **Identification:** identifiedBy: Denis Gradinarov; dateIdentified: 2025; **Event:** samplingProtocol: on flowers; verbatimEventDate: 23.vii.2021; habitat: meadows, edge of oak forest
- **Type status:** Other material. **Occurrence:** catalogNumber: BFUS-COL000621; recordedBy: Denis Gradinarov & Yana Petrova; individualCount: 1; sex: female; occurrenceID: 7E51DE45-94E3-5152-9A4E-1981F1BE021D; **Location:** country: Bulgaria; stateProvince: Haskovo; municipality: Topolovgrad; locality: Sakar Mountains, SE of Ustrem Village, the road to Radovets Village; verbatimElevation: 203 m; decimalLatitude: 41.975300; decimalLongitude: 26.483550; geodeticDatum: WGS84; **Identification:** identifiedBy: Denis Gradinarov; dateIdentified: 2025; **Event:** samplingProtocol: on flowers; verbatimEventDate: 30.vi.2022; habitat: meadows, edge of mixed forest
- **Type status:** Other material. **Occurrence:** catalogNumber: BFUS-COL000622; recordedBy: Denis Gradinarov & Yana Petrova; individualCount: 1; sex: female; occurrenceID: 42431046-17B6-593D-996C-EAFBD1B6A22C; **Location:** country: Bulgaria; stateProvince: Haskovo; municipality: Topolovgrad; locality: Sakar Mountains, SE of Ustrem Village, the road to Radovets Village; verbatimElevation: 203 m; decimalLatitude: 41.975300; decimalLongitude: 26.483550; geodeticDatum: WGS84; **Identification:** identifiedBy: Denis Gradinarov; dateIdentified: 2025; **Event:** samplingProtocol: on flowers; verbatimEventDate: 30.vi.2022; habitat: meadows, edge of mixed forest
- **Type status:** Other material. **Occurrence:** catalogNumber: BFUS-COL000623; recordedBy: Denis Gradinarov; individualCount: 1; sex: male; occurrenceID: DDB57FAE-583C-5FBF-8A90-D676F8857A5C; **Location:** country: Bulgaria; stateProvince: Haskovo; municipality: Topolovgrad; locality: Sakar Mountains, SW of Sinapovo Village; verbatimElevation: 221 m; decimalLatitude: 42.115000; decimalLongitude: 26.412783; geodeticDatum: WGS84; **Identification:** identifiedBy: Denis Gradinarov; dateIdentified: 2025; **Event:** samplingProtocol: on Apiaceae; verbatimEventDate: 24.v.2023; habitat: meadows, edge of mixed forest
- **Type status:** Other material. **Occurrence:** catalogNumber: BFUS-COL000624; recordedBy: Denis Gradinarov; individualCount: 1; sex: male; occurrenceID: 45E088E6-E406-5836-BF79-ABB8E11BE756; **Location:** country: Bulgaria; stateProvince: Haskovo; municipality: Topolovgrad; locality: Sakar Mountains, SW of Sinapovo Village; verbatimElevation: 221 m; decimalLatitude: 42.115000; decimalLongitude: 26.412783; geodeticDatum: WGS84; **Identification:** identifiedBy: Denis Gradinarov; dateIdentified: 2025; **Event:** samplingProtocol: on Apiaceae; verbatimEventDate: 24.v.2023; habitat: meadows, edge of mixed forest
- **Type status:** Other material. **Occurrence:** catalogNumber: BFUS-COL000625; recordedBy: Denis Gradinarov; individualCount: 1; sex: male; occurrenceID: 0FD3B5B2-743E-54B5-AEE7-DACA2BB9E616; **Location:** country: Bulgaria; stateProvince: Haskovo; municipality: Topolovgrad; locality: Sakar Mountains, SW of Sinapovo Village; verbatimElevation: 221 m; decimalLatitude: 42.115000; decimalLongitude: 26.412783; geodeticDatum: WGS84; **Identification:** identifiedBy: Denis Gradinarov; dateIdentified: 2025; **Event:** samplingProtocol: on Apiaceae; verbatimEventDate: 24.v.2023; habitat: meadows, edge of mixed forest
- **Type status:** Other material. **Occurrence:** catalogNumber: BFUS-COL000626; recordedBy: Denis Gradinarov; individualCount: 1; sex: female; occurrenceID: 47009801-F9C2-5092-A38C-B21C6D8ED0A3; **Location:** country: Bulgaria; stateProvince: Haskovo; municipality: Topolovgrad; locality: Sakar Mountains, SW of Sinapovo Village; verbatimElevation: 221 m; decimalLatitude: 42.115000; decimalLongitude: 26.412783; geodeticDatum: WGS84; **Identification:** identifiedBy: Denis Gradinarov; dateIdentified: 2025; **Event:** samplingProtocol: on Apiaceae; verbatimEventDate: 24.v.2023; habitat: meadows, edge of mixed forest
- **Type status:** Other material. **Occurrence:** catalogNumber: BFUS-COL000627; recordedBy: Denis Gradinarov; individualCount: 1; sex: female; occurrenceID: 9D6BF8E7-EF8F-5A99-9100-4999803E9E68; **Location:** country: Bulgaria; stateProvince: Haskovo; municipality: Topolovgrad; locality: Sakar Mountains, SW of Sinapovo Village; verbatimElevation: 221 m; decimalLatitude: 42.115000; decimalLongitude: 26.412783; geodeticDatum: WGS84; **Identification:** identifiedBy: Denis Gradinarov; dateIdentified: 2025; **Event:** samplingProtocol: on Apiaceae; verbatimEventDate: 24.v.2023; habitat: meadows, edge of mixed forest
- **Type status:** Other material. **Occurrence:** catalogNumber: BFUS-COL000628; recordedBy: Denis Gradinarov; individualCount: 1; sex: female; occurrenceID: 8D0EFC7A-32DB-5403-AF2C-E91E2EAA67D6; **Location:** country: Bulgaria; stateProvince: Haskovo; municipality: Topolovgrad; locality: Sakar Mountains, SW of Sinapovo Village; verbatimElevation: 221 m; decimalLatitude: 42.115000; decimalLongitude: 26.412783; geodeticDatum: WGS84; **Identification:** identifiedBy: Denis Gradinarov; dateIdentified: 2025; **Event:** samplingProtocol: on Apiaceae; verbatimEventDate: 24.v.2023; habitat: meadows, edge of mixed forest

#### Description

**Figure 2.**
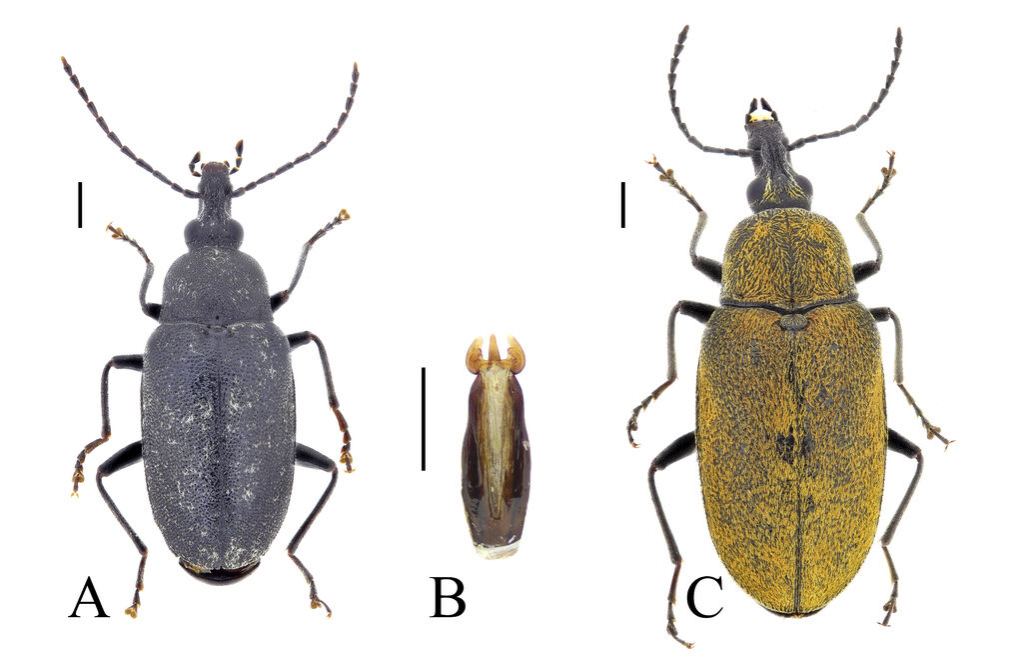
Habitus of *Mycterus curculioides* (Fabricius, 1781) from Bulgaria: **A** male from Sakar Mountains (BFUS-COL000617); **B** aedeagus of the same specimen; **C** female from Rila Mountains (BFUS-COL000615). Scale bars: 1 mm (A, C); 0.5 mm (B).

The species is characterised by a long, widened anteriorly rostrum and by its black legs and antennae (Fig. 2). The second abdominal sternite in males has a distinctly raised sex patch with dense pubescence. Aedeagus is short, with thick parameres (Iablokoff-Khnzorian 1985) (Fig. 2).

### Pytho depressus

(Linnaeus, 1767)

9991262D-8468-5D87-B9E2-0EFD990F54F0

#### Materials

- **Type status:** Other material. **Occurrence:** catalogNumber: BFUS-COL000629; recordedBy: Denis Gradinarov; individualCount: 1; sex: male; occurrenceID: 04D5CBA8-62BB-55E7-BFED-47556413D254; **Location:** country: Bulgaria; stateProvince: Sofia City; municipality: Stolichna; locality: Vitosha Mountains, NW of Zheleznitsa Village; verbatimElevation: 1226 m; decimalLatitude: 42.538033; decimalLongitude: 23.346050; geodeticDatum: WGS84; **Identification:** identifiedBy: Denis Gradinarov; dateIdentified: 2025; **Event:** samplingProtocol: under bark of *Pinus sylvestris*; verbatimEventDate: 13.iv.2022; habitat: pine forest
- **Type status:** Other material. **Occurrence:** catalogNumber: BFUS-COL000630; recordedBy: Denis Gradinarov; individualCount: 1; sex: female; occurrenceID: 68B10EA6-00F4-5428-BE3C-63F9D164C5EB; **Location:** country: Bulgaria; stateProvince: Sofia City; municipality: Stolichna; locality: Vitosha Mountains, near to Moreni Hotel; verbatimElevation: 1731 m; decimalLatitude: 42.592383; decimalLongitude: 23.297317; geodeticDatum: WGS84; **Identification:** identifiedBy: Denis Gradinarov; dateIdentified: 2025; **Event:** samplingProtocol: under bark of *Picea abies*; verbatimEventDate: 14.v.2022; habitat: spruce forest
- **Type status:** Other material. **Occurrence:** catalogNumber: BFUS-COL000631; recordedBy: Denis Gradinarov; individualCount: 1; sex: female; occurrenceID: 115396E7-EB5B-5DFB-8CA7-A54AF9A4BF40; **Location:** country: Bulgaria; stateProvince: Sofia City; municipality: Stolichna; locality: Vitosha Mountains, near to Moreni Hotel; verbatimElevation: 1731 m; decimalLatitude: 42.592383; decimalLongitude: 23.297317; geodeticDatum: WGS84; **Identification:** identifiedBy: Denis Gradinarov; dateIdentified: 2025; **Event:** samplingProtocol: under bark of *Picea abies*; verbatimEventDate: 14.v.2022; habitat: spruce forest

#### Description

**Figure 3.**
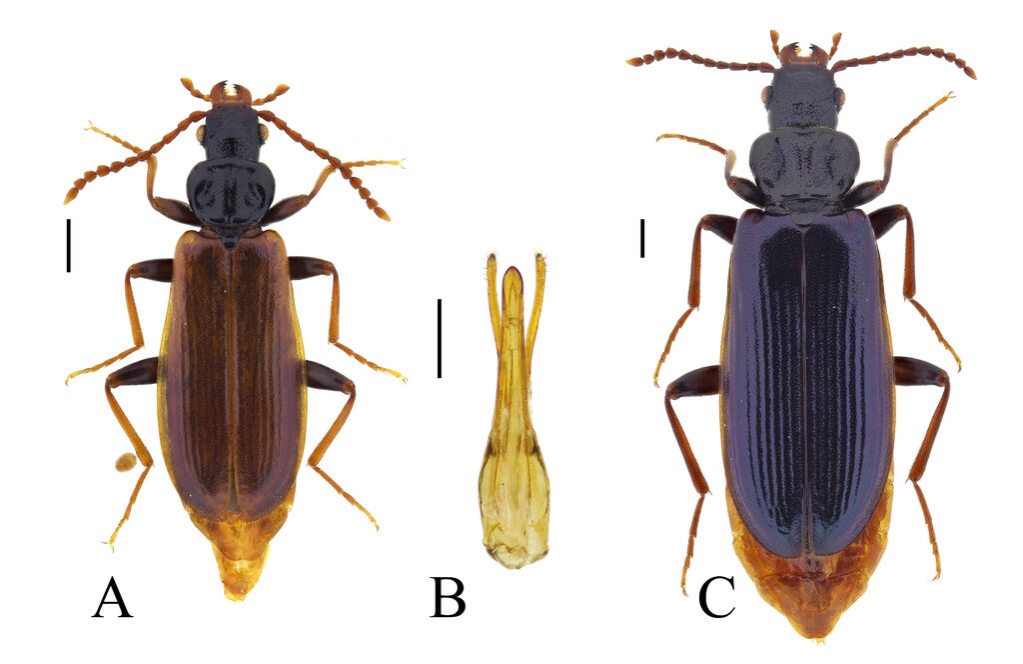
Habitus of *Pytho depressus* (Linnaeus, 1767) from Vitosha Mountains, Bulgaria: **A** male (BFUS-COL000629); **B** aedeagus of the same specimen; **C** female (BFUS-COL000630). Scale bars: 1 mm (A, C); 0.5 mm (B).

*Pytho depressus* can be distinguished from the closely related Nearctic species *P. americanus* Kirby, 1837 by the aedeagus with apicale approximately 1.5 times length of basale (apicale and basale subequal in length in *P. americanus*) (Pollock 1991), from the other Palaearctic species of the genus – in particular, by the metallic lustre of the elytra, shape of the pronotum (slightly arcuate lateral margins and greatest width anterior of midlength), variable colour of the head, pronotum and elytra, as well as by aedeagus morphology (Pollock 1991, Háva and Zahradník 2021, Chittaro et al. 2023) (Fig. 3).

### Salpingus planirostris

(Fabricius, 1787)

0447C1AF-7F45-588B-A560-E4F1DD1E265B

#### Materials

- **Type status:** Other material. **Occurrence:** catalogNumber: BFUS-COL000632; recordedBy: Ognyan Sivilov & Hristina Hristova; individualCount: 1; sex: male; occurrenceID: ABFCF611-C37A-505D-A503-A0B17D59138D; **Location:** country: Bulgaria; stateProvince: Lovech; municipality: Troyan; locality: Central Stara Planina Mountains, SW of Chiflik Village, near Beli Osam River; verbatimElevation: 760 m; decimalLatitude: 42.823450; decimalLongitude: 24.540833; geodeticDatum: WGS84; **Identification:** identifiedBy: Denis Gradinarov; dateIdentified: 2025; **Event:** samplingProtocol: flight interception trap; verbatimEventDate: 14.iv.2020–13.v.2020; habitat: beech forest (*Fagus sylvatica* L.) with solitary trees of *Populus tremula* L., *Abies alba* Mill. and other tree species
- **Type status:** Other material. **Occurrence:** catalogNumber: BFUS-COL000633; recordedBy: Ognyan Sivilov & Hristina Hristova; individualCount: 1; sex: female; occurrenceID: DF2613B5-840B-5756-8740-5EBFAC09FEE6; **Location:** country: Bulgaria; stateProvince: Lovech; municipality: Troyan; locality: Central Stara Planina Mountains, SW of Chiflik Village, near Beli Osam River; verbatimElevation: 760 m; decimalLatitude: 42.823450; decimalLongitude: 24.540833; geodeticDatum: WGS84; **Identification:** identifiedBy: Denis Gradinarov; dateIdentified: 2025; **Event:** samplingProtocol: flight interception trap; verbatimEventDate: 13.v.2020–28.v.2020; habitat: beech forest (*Fagus sylvatica* L.) with solitary trees of *Populus tremula* L., *Abies alba* Mill. and other tree species
- **Type status:** Other material. **Occurrence:** catalogNumber: BFUS-COL000634; recordedBy: Ognyan Sivilov & Hristina Hristova; individualCount: 1; sex: female; occurrenceID: C697902A-5D55-55FA-84C3-F3297247BA1C; **Location:** country: Bulgaria; stateProvince: Lovech; municipality: Troyan; locality: Central Stara Planina Mountains, SW of Chiflik Village, near Beli Osam River; verbatimElevation: 760 m; decimalLatitude: 42.823450; decimalLongitude: 24.540833; geodeticDatum: WGS84; **Identification:** identifiedBy: Denis Gradinarov; dateIdentified: 2025; **Event:** samplingProtocol: flight interception trap; verbatimEventDate: 04.vii.2020–30.vii.2020; habitat: beech forest (*Fagus sylvatica* L.) with solitary trees of *Populus tremula* L., *Abies alba* Mill. and other tree species
- **Type status:** Other material. **Occurrence:** catalogNumber: BFUS-COL000646; recordedBy: Ognyan Sivilov & Hristina Hristova; individualCount: 1; sex: specimen; occurrenceID: D4557628-3B2B-548C-B6FF-4551D7F725EA; **Location:** country: Bulgaria; stateProvince: Lovech; municipality: Troyan; locality: Central Stara Planina Mountains, SW of Chiflik Village, near Beli Osam River; verbatimElevation: 760 m; decimalLatitude: 42.823450; decimalLongitude: 24.540833; geodeticDatum: WGS84; **Identification:** identifiedBy: Denis Gradinarov; dateIdentified: 2025; **Event:** samplingProtocol: flight interception trap; verbatimEventDate: 04.vii.2020–30.vii.2020; habitat: beech forest (*Fagus sylvatica* L.) with solitary trees of *Populus tremula* L., *Abies alba* Mill. and other tree species

#### Description

**Figure 4.**
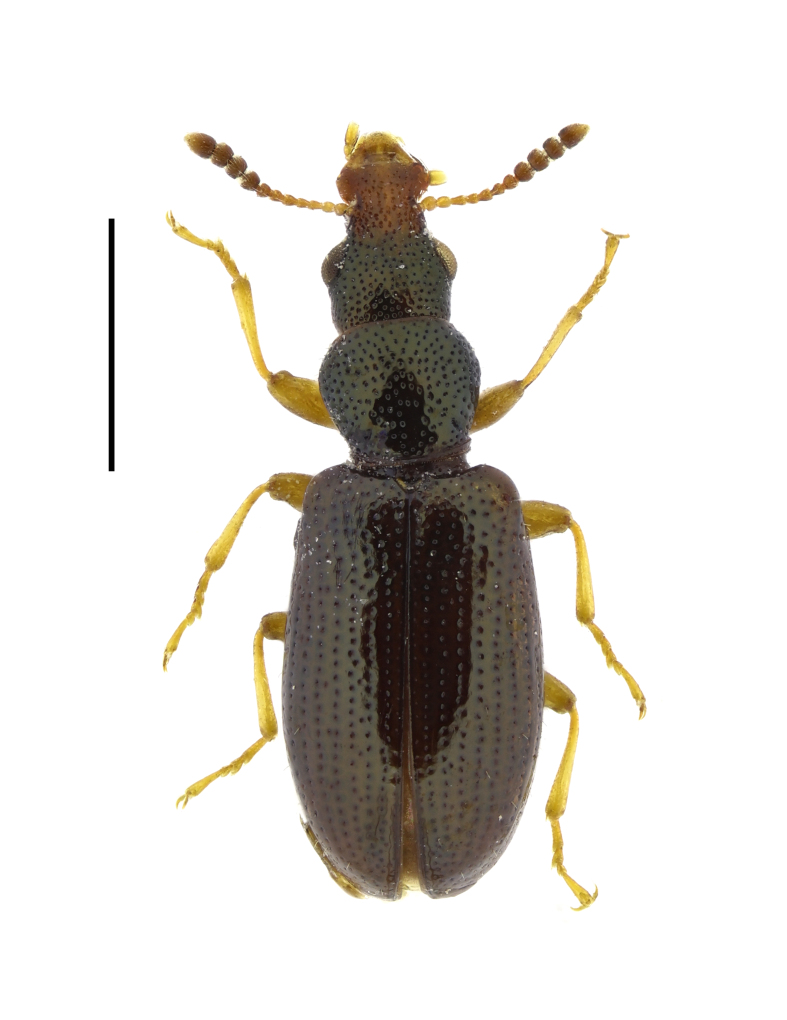
Habitus of *Salpingus planirostris* (Fabricius, 1787) from Central Stara Planina Mountains, Bulgaria (female, BFUS-COL000634). Scale bar: 1 mm.

The species is characterised by its elongated and broad rostrum and dorsal body colour dark brown with bronze sheen (Fig. 4). Rostrum, legs and antennae yellowish-brown, antennal club darkened. Elytra with weak transverse depression on discal region. Antenna with distinct club of four antennomeres (Iablokoff-Khnzorian 1985, Barnouin and Zagatti 2017, Viñolas et al. 2021).

### Salpingus ruficollis

(Linnaeus, 1760)

F2000DC0-A8CD-5B4B-9EFA-26F054960323

#### Materials

- **Type status:** Other material. **Occurrence:** catalogNumber: BFUS-COL000635; recordedBy: Yana Petrova; individualCount: 1; sex: female; occurrenceID: 4F70E7F0-E17A-5A00-829C-1FF52815CCA2; **Location:** country: Bulgaria; stateProvince: Stara Zagora; municipality: Bratya Daskalovi; locality: Sarnena Gora Mountains, 3 km NW Gorno Novo Selo Village, near Kaleto Place; verbatimElevation: 800 m; decimalLatitude: 42.474183; decimalLongitude: 25.222067; geodeticDatum: WGS84; **Identification:** identifiedBy: Denis Gradinarov; dateIdentified: 2025; **Event:** samplingProtocol: under bark; verbatimEventDate: 24.ix.2018; habitat: edge of deciduous forest
- **Type status:** Other material. **Occurrence:** catalogNumber: BFUS-COL000636; recordedBy: Yana Petrova; individualCount: 1; sex: female; occurrenceID: 284DFA35-C8C6-54D4-90C1-8A23493634CE; **Location:** country: Bulgaria; stateProvince: Stara Zagora; municipality: Bratya Daskalovi; locality: Sarnena Gora Mountains, 3 km NW Gorno Novo Selo Village, near Kaleto Place; verbatimElevation: 800 m; decimalLatitude: 42.474183; decimalLongitude: 25.222067; geodeticDatum: WGS84; **Identification:** identifiedBy: Denis Gradinarov; dateIdentified: 2025; **Event:** samplingProtocol: under bark; verbatimEventDate: 24.ix.2018; habitat: edge of deciduous forest
- **Type status:** Other material. **Occurrence:** catalogNumber: BFUS-COL000637; recordedBy: Yana Petrova; individualCount: 1; sex: specimen; occurrenceID: 8C3A56C7-22BB-5FCB-8CDF-E8C29462EF7A; **Location:** country: Bulgaria; stateProvince: Stara Zagora; municipality: Bratya Daskalovi; locality: Sarnena Gora Mountains, 3 km NW Gorno Novo Selo Village, near Kaleto Place; verbatimElevation: 800 m; decimalLatitude: 42.474183; decimalLongitude: 25.222067; geodeticDatum: WGS84; **Identification:** identifiedBy: Denis Gradinarov; dateIdentified: 2025; **Event:** samplingProtocol: under bark; verbatimEventDate: 24.ix.2018; habitat: edge of deciduous forest
- **Type status:** Other material. **Occurrence:** catalogNumber: BFUS-COL000638; recordedBy: Ognyan Sivilov & Hristina Hristova; individualCount: 1; sex: female; occurrenceID: 9AF8714E-7C15-53B1-BF3E-515CAED62536; **Location:** country: Bulgaria; stateProvince: Kyustendil; municipality: Rila; locality: Rila Mountains, near Iliyna Reka River; verbatimElevation: 1325 m; decimalLatitude: 42.091967; decimalLongitude: 23.385017; geodeticDatum: WGS84; **Identification:** identifiedBy: Denis Gradinarov; dateIdentified: 2025; **Event:** samplingProtocol: flight interception trap; verbatimEventDate: 25.vi.2020; habitat: deciduous forest

#### Description

**Figure 5.**
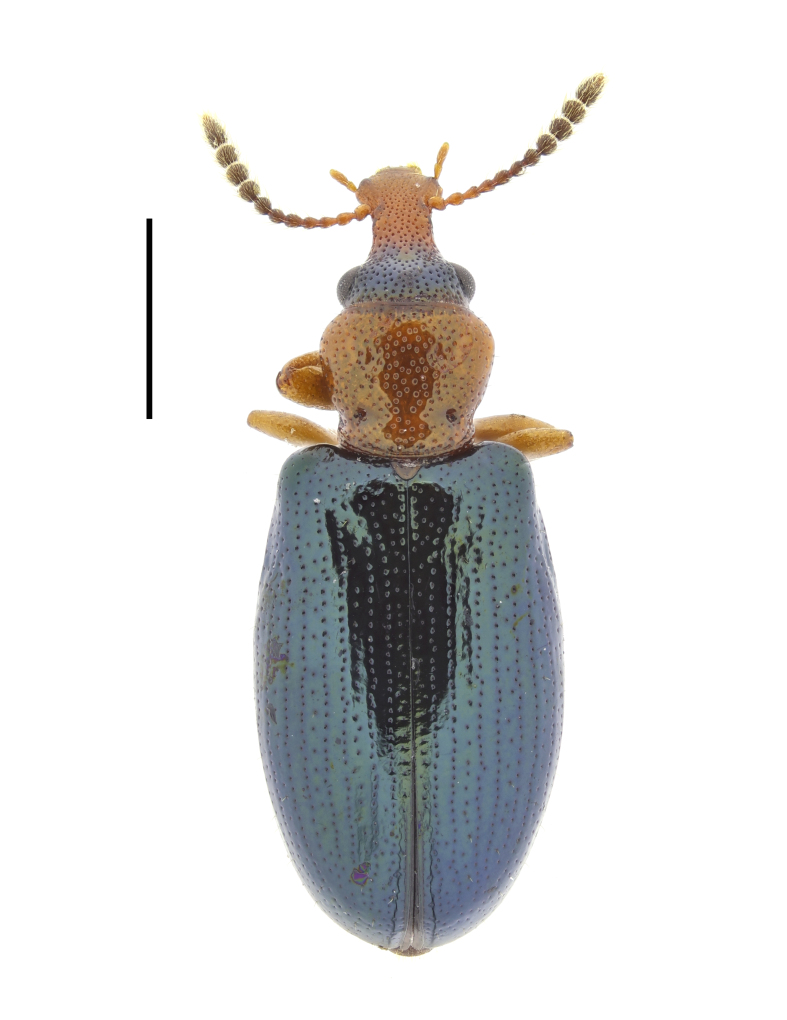
Habitus of *Salpingus ruficollis* (Linnaeus, 1760) from Sarnena Gora Mountains, Bulgaria (female, BFUS-COL000635). Scale bar: 1 mm.

The species is characterised by its elongated (3 times as long as wide at the base) and anteriorly very broad rostrum, brownish-red head with a black patch between the eyes, brownish-red pronotum and blue or green metallic elytra without transverse depressions (Fig. 5). The antennae have an indistinct club of five antennomeres (Iablokoff-Khnzorian 1985, Barnouin and Zagatti 2017, Viñolas et al. 2021).

### Vincenzellus ruficollis

(Panzer, 1794)

43745620-A664-500A-99F3-E45CB1D20EF6

#### Materials

- **Type status:** Other material. **Occurrence:** catalogNumber: BFUS-COL000639; recordedBy: Ognyan Sivilov & Hristina Hristova; individualCount: 1; sex: male; occurrenceID: 681B374A-6F6B-57B1-8659-51C21E59FF10; **Location:** country: Bulgaria; stateProvince: Lovech; municipality: Troyan; locality: Central Stara Planina Mountains, SW of Chiflik Village, near Beli Osam River; verbatimElevation: 760 m; decimalLatitude: 42.823450; decimalLongitude: 24.540833; geodeticDatum: WGS84; **Identification:** identifiedBy: Denis Gradinarov; dateIdentified: 2025; **Event:** samplingProtocol: flight interception trap; verbatimEventDate: 13.v.2020–28.v.2020; habitat: beech forest (*Fagus sylvatica* L.) with solitary trees of *Populus tremula* L., *Abies alba* Mill. and other tree species
- **Type status:** Other material. **Occurrence:** catalogNumber: BFUS-COL000640; recordedBy: Ognyan Sivilov & Hristina Hristova; individualCount: 1; sex: female; occurrenceID: 76641DB9-93DE-5949-B668-F3A3C7AFE26C; **Location:** country: Bulgaria; stateProvince: Lovech; municipality: Troyan; locality: Central Stara Planina Mountains, SW of Chiflik Village, near Beli Osam River; verbatimElevation: 760 m; decimalLatitude: 42.823450; decimalLongitude: 24.540833; geodeticDatum: WGS84; **Identification:** identifiedBy: Denis Gradinarov; dateIdentified: 2025; **Event:** samplingProtocol: flight interception trap; verbatimEventDate: 13.v.2020–28.v.2020; habitat: beech forest (*Fagus sylvatica* L.) with solitary trees of *Populus tremula* L., *Abies alba* Mill. and other tree species
- **Type status:** Other material. **Occurrence:** catalogNumber: BFUS-COL000641; recordedBy: Ognyan Sivilov & Hristina Hristova; individualCount: 1; sex: female; occurrenceID: 7684C949-C06C-5436-94C0-B1A2D25A9242; **Location:** country: Bulgaria; stateProvince: Lovech; municipality: Troyan; locality: Central Stara Planina Mountains, SW of Chiflik Village, near Beli Osam River; verbatimElevation: 760 m; decimalLatitude: 42.823450; decimalLongitude: 24.540833; geodeticDatum: WGS84; **Identification:** identifiedBy: Denis Gradinarov; dateIdentified: 2025; **Event:** samplingProtocol: flight interception trap; verbatimEventDate: 13.v.2020–28.v.2020; habitat: beech forest (*Fagus sylvatica* L.) with solitary trees of *Populus tremula* L., *Abies alba* Mill. and other tree species
- **Type status:** Other material. **Occurrence:** catalogNumber: BFUS-COL000642; recordedBy: Ognyan Sivilov & Hristina Hristova; individualCount: 1; sex: female; occurrenceID: 30D8D554-E6DB-5B16-AB51-995A389CC413; **Location:** country: Bulgaria; stateProvince: Lovech; municipality: Troyan; locality: Central Stara Planina Mountains, SW of Chiflik Village, near Beli Osam River; verbatimElevation: 760 m; decimalLatitude: 42.823450; decimalLongitude: 24.540833; geodeticDatum: WGS84; **Identification:** identifiedBy: Denis Gradinarov; dateIdentified: 2025; **Event:** samplingProtocol: flight interception trap; verbatimEventDate: 13.v.2020–28.v.2020; habitat: beech forest (*Fagus sylvatica* L.) with solitary trees of *Populus tremula* L., *Abies alba* Mill. and other tree species

#### Description

**Figure 6.**
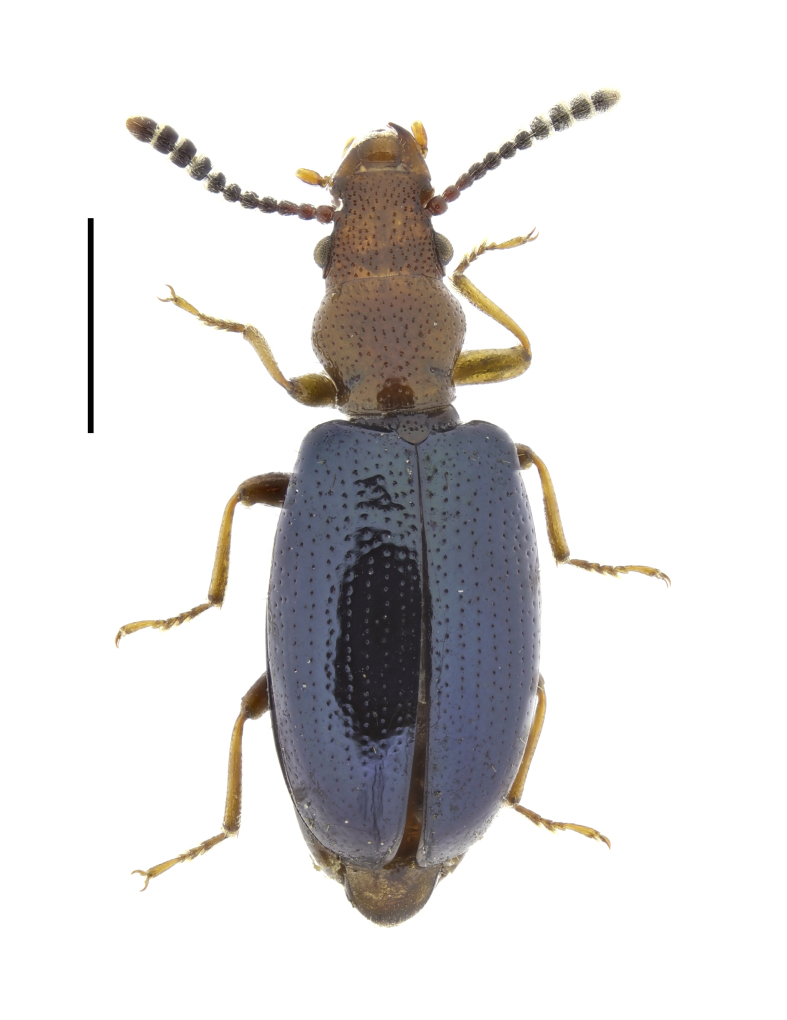
Habitus of *Vincenzellus ruficollis* (Panzer, 1794) from Central Stara Planina Mountains, Bulgaria (male, BFUS-COL000639). Scale bar: 1 mm.

This species is characterised by its relatively short and very broad rostrum, bicoloured body with brownish-red head and pronotum and metallic blue elytra (Fig. 6). The pronotum has distinct paired basal depressions. The antenna has an indistinct club of five antennomeres, colour reddish-brown with club generally darker (Barnouin and Zagatti 2017, Viñolas et al. 2021).

## Checklists

### Checklist of the families Mycteridae, Pythidae and Salpingidae of Bulgaria

#### Coleoptera

Linnaeus, 1758

4F109BD3-B37B-5099-AC25-AE4EE16B437E

#### Polyphaga

Emery, 1886

BA347D3E-8C57-5B72-BC01-18A769C6479B

#### Tenebrionoidea

Latreille, 1802

D4A57661-19C0-5C3B-B65E-A38A4BB08001

#### Mycteridae

Oken, 1843

038D5E3D-055F-5FEB-83CE-28A713A6E742

#### Mycterinae

Oken, 1843

391EB109-27BB-5DCB-A952-DC2228E37B57

#### Mycterus

Clairville, 1798

021467BF-9E93-539D-8ACC-740E56B9FC24

#### Eutryptes

Gistel, 1856

3F9D9D56-033B-52BD-B381-067C99C83F96

#### Mycterus (Eutryptes) tibialis

Küster, 1850

D7842802-4F75-5232-B4A7-737082936D6D

##### Distribution

Austria, Bosnia and Herzegovina, Croatia, Czecha, Germany, Greece, Hungary, Italy, Slovakia, South European Territory of Russia, Switzerland, Ukraine, Serbia and Montenegro, both the European and Asian territories of Turkiye (Löbl et al. 2020), Romania (Jaquet 1901) and Bulgaria (Joakimov 1904, Nedelkov 1909, Roubal 1931, Panin 1941, Angelov 1968a, Guéorguiev and Ljubomirov 2009).

##### Notes

In Bulgaria, the species has been reported from Haskovo (Joakimov 1904: 43), Lyulin Mountains (Nedelkov (1909): 8, as *Mycterus umbellatarum* Fab.) [one female revised in NMNHS], Pirin Mountains (Roubal 1931: 453), Batova River Valley in the vicinty of Ecréné [= Kranevo] Village (Panin 1941: 552), Murgash Peak in Western Stara Planina Mountains (Angelov 1968a: 145) and Maleshevska Planina Mountains (Guéorguiev and Ljubomirov 2009: 255).

#### Mycterus

Clairville, 1798

3122C529-B1E4-5EDE-ABB5-E708B5C850B6

#### Mycterus (Mycterus) curculioides

(Fabricius, 1781)

D09177FA-77C5-5677-BDF3-55FFDFCEC61D

##### Distribution

Austria, Croatia, Czechia, France, Great Britain, Germany, Greece, Hungary, Italy, Latvia, Poland, Portugal, Romania, Slovakia, Spain, Switzerland, Ukraine, Central and South European Territory of Russia, Azerbaijan, Cyprus, Israel, Lebanon, Asian territory of Turkiye, Morocco, Tunisia (Löbl et al. 2020) and Bulgaria (Gradinarov and Petrova 2024).

##### Notes

In Bulgaria, the species was recorded only from Sakar Mountains, vicinity of Ustrem Village (Gradinarov and Petrova (2024): 120, as *Mycterus curculionoides* [sic!] (Fabricius, 1781)).

#### Pythidae

Solier, 1834

7E92FD6C-37B0-50BE-A7D0-ED1E99EF2CE4

#### Pytho

Latreille, 1796

53C97121-7F96-5CBF-A35E-EAEC575FAC76

#### Pytho depressus

(Linnaeus, 1767)

217E43AA-C686-500B-9110-6DB133AE2DA9

##### Distribution

Austria, Bosnia and Herzegovina, Belarus, Czechia, Denmark, Estonia, Finland, France, Great Britain, Germany, Georgia, Hungary, Italy, Kazakhstan, Latvia, Lithuania, The Netherlands, Norway, Poland, Romania, Russia (North, Central and South European Territory, East and West Siberia, Far East), Slovakia, Spain, Sweden, Switzerland, Ukraine, (Pollock and Iwan 2020), Bulgaria (Roubal 1931, Kovács et al. 2011), China: Heilongjiang Province (Painter et al. 2007), China: Xinjiang Province (Háva and Zahradník 2021), Moldova, Serbia (Jaskuła et al. 2024) and Greece (Gkourogiannis et al. 2025).

##### Notes

In Bulgaria, the species is known from Rila Mountains (without exact locality and from the vicinity of Tiha Rila hut) (Roubal 1931: 453; Hubenov et al. 2000: 569; Kovács et al. 2011: 41), Pirin Mountains (Sandanski) and Vitosha Mountains (Balabana locality above Boyana) (Kovács et al. 2011: 41).

#### Salpingidae

Leach, 1815

CCBE7A3B-34CF-5863-BE6D-DCCA8337B72D

#### Salpinginae

Leach, 1815

793A84BF-4943-55D7-A9C4-F71320D45CDF

#### Lissodema

Curtis, 1833

AB243885-04B9-54CE-9A92-050CEB9C4873

#### Lissodema denticollis

(Gyllenhal, 1813)

8A3AFAF7-92DA-5445-BD55-8CD28A4DFE92

##### Distribution

Armenia, Austria, Belgium, Bosnia and Herzegovina, Bulgaria, Croatia, Czechia, Denmark, France, Great Britain, Germany, Greece, Hungary, Ireland, Italy, Luxembourg, The Netherlands, Poland, Romania, Slovakia, Spain, Sweden, Switzerland, South European Territory of Russia and “Caucasus” (Pollock et al. 2020).

##### Notes

In Bulgaria, the species is known only from Maglige [= Maglizh, South-Central Bulgaria] (Horion (1956): 35, as *Lissodema quadripustulata* Marsh.).

#### Salpingus

Illiger, 1802

A5912AB0-FC1C-54AF-AF42-25B79C867619

#### Salpingus planirostris

(Fabricius, 1787)

E85CED8D-9BE5-571D-98AE-3E16E0626236

##### Distribution

Andorra, Belgium, Bulgaria, Belarus, Croatia, Czechia, Denmark, Estonia, Finland, France, Great Britain, Germany, Hungary, Ireland, Italy, Latvia, Lithuania, Luxembourg, The Netherlands, Norway, Poland, Portugal, Romania, Spain, Sweden, North, Central and South European Territory of Russia, “Caucasus”, Morocco and Tunisia (Pollock et al. 2020).

##### Notes

In Bulgaria, the species has been previously reported only from Western Rhodopes Mountains (next to Lepenitsa Cave) (Guéorguiev and Bekchiev 2009: 42).

#### Salpingus ruficollis

(Linnaeus, 1760)

AD0D7FEC-94AC-55F8-B317-EEAF042DDFF1

##### Distribution

Austria, Belarus, Croatia, Czechia, Denmark, Estonia, Finland, France, Germany, Hungary, Ireland, Italy, Latvia, Lithuania, Luxembourg, The Netherlands, Norway, Poland, Romania, Russia (North and Central European Territory), Slovakia, Slovenia, Spain, Sweden, Switzerland, Ukraine (Pollock et al. 2020), Bulgaria (Angelov 1968b), Greece (Kakiopoulos and Demetriou 2022) and South European Territory of Russia (Volodchenko and Sazhnev 2023).

##### Notes

In Bulgaria, the species has been previously reported only from Western Stara Planina Mts, Izdremets Peak (Angelov (1968b): 99, as *Rhinosimus ruficollis* (L.)). The species was omitted in the country list for Bulgaria in the second edition of the Catalogue of Palaearctic Coleoptera (Pollock et al. 2020).

#### Vincenzellus

Reitter, 1911

EB30E882-5006-5C33-B75E-3F014D640825

#### Vincenzellus ruficollis

(Panzer, 1794)

2091919F-0760-5C9E-BD06-282C9B2031FB

##### Distribution

Austria, Belgium, Bosnia and Herzegovina, Bulgaria, Croatia, Czechia, Denmark, France, Germany, Hungary, Ireland, Italy, Luxembourg, The Netherlands, Poland, Romania, Slovakia, Spain, Sweden, Switzerland, Ukraine, Serbia and Montenegro (Pollock et al. 2020), Russia (Central and South European Territory) (Nikitsky 2016), Greece (Kakiopoulos and Demetriou 2022) and Iran (Mazandaran Province) (Farashiani et al. 2022).

##### Notes

In Bulgaria, the species has been previously reported from Kritschin [= Krichim, town in South-Central Bulgaria] (Horion 1956: 45) and from Belasitsa Mountains, above Kamena Village (Guéorguiev and Bekchiev 2009: 42).

## Discussion

**Figure 7.**
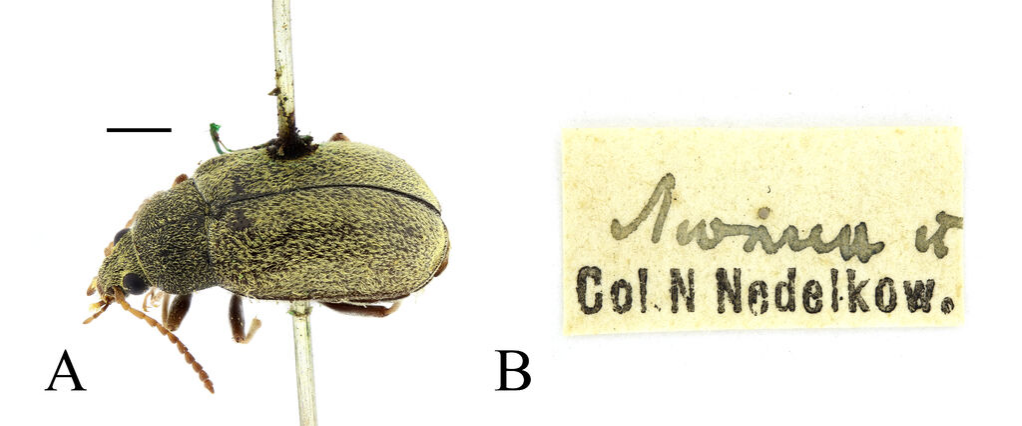
Specimen of *Mycterus tibialis* Küster, 1850 from Lyulin Mountains, preserved in the historical collection of Nikola Nedelkov, NMNHS. **A** pinned specimen (female); **B** original label. Scale bar: 1 mm (A).

*Mycterus tibialis* is known from Central, Southern (Apennine and Balkan Peninsulas) and Eastern Europe, as well as from the Asian part of Turkiye (Löbl et al. 2020). In the second edition of the Catalogue of Palaearctic Coleoptera (Löbl et al. 2020), the species is omitted for Romania (Jaquet (1901): 492, record from Mangalia) and for Bulgaria. *Mycterus tibialis* was reported as new to Bulgaria by Angelov (1968a) from Western Stara Planina Mountains (Murgash Peak). However, earlier, Joakimov (1904) reported this species from Haskovo, Roubal (1931) – from Pirin Mountains and Panin (1941) – from the Batova River Valley. Recently, the species was also found in Maleshevska Planina Mountains (Guéorguiev and Ljubomirov 2009). The old record of the Mediterranean species *Mycterus umbellatarum* (Fabricius, 1787) from Lyulin Mountain (SW Bulgaria) (Nedelkov 1909) actually refer to *Mycterus tibialis*. In the systematic collection of Nikola Nedelkov housed in NMNHS, only two specimens of the genus *Mycterus*, classified within the family Pythidae, are preserved. The first has a label “Burgas [handwritten in Bulgarian]/Col. N Nedelkow.” and a handwritten identification label “*Mycterus*/*umbellatarum* F.” The second one lacks an individual identification label, but the locality on the main label corresponds to that in Nedelkov's publication – Lyulin [Mts] (handwritten in Bulgarian) (Fig. 7). Both specimens, after revision of the identification, actually turned out to be females of *Mycterus tibialis*. The report of *M. articulatus* Reitter, 1911 from Sliven, south-eastern Bulgaria, by Roubal (1932): 129 probably aslo concerns *Mycterus tibialis*. The last two species are closely related and similar in appearance, but *M. articulatus* is known from Transcaucasia (Iablokoff-Khnzorian 1985, Löbl et al. 2020) and has not been reported from Europe since Roubal. Here, we record *M. tibialis* from a number of localities from the Black Sea Coast, Western, Middle and Eastern Danubian Plain, Eastern Rhodopes Mountains, Strandzha Mountains, Sakar Mountains, Derventski Vazvisheniya Hills and the Bulgarian part of the Falakro Mountains. Our and literature data indicate that *M. tibialis* is a widespread and rather common species in Bulgaria.

*Mycterus curculioides* is fairly widespread in Europe, excluding the northern regions (Löbl et al. 2020). In Bulgaria, the species has been recently reported from Sakar Mountain as a prey item of *Trichodes crabroniformis* (Fabricius, 1787) (Coleoptera, Cleridae) (Gradinarov and Petrova 2024). Surprisingly, we did not find any earlier reports of this species from Bulgaria. In the Sakar region, this species is rather common and, here, we report it from a number of localities. In neighbouring regions – Derventski Vazvisheniya Hills, Strandzha and Eastern Rhodopes, we have not found this species, but only *M. tibialis*. The locality of *M. curculioides* from Rila (ca. 1150 m a.s.l.) indicates that the species in Bulgaria occurs in a wide elevational range, which has been observed in other countries in southern Europe (Verdugo and Valladares 2012, Obregón and Murillo 2013, Terzani and Ceccolini 2015). Further studies are needed to clarify the distribution of this species in Bulgaria and its habitat requirements.

*Pytho depressus* is the most widespread Palaearctic species of the genus (Pollock 1991, Pollock and Iwan 2020). The species is distributed from Scandinavia to eastern Russia and northern China, in Europe with southern extensions in mountainous regions with coniferous forests (Pollock 1991, Pollock and Iwan 2020, Painter et al. 2007, Háva and Zahradník 2021, Jaskuła et al. 2024, Gkourogiannis et al. 2025). The species was reported as new to Bulgaria by Kovács et al. (2011), but in fact, the first country record has to be referred to Roubal (1931). *Pytho depressus* is omitted for Bulgaria in the second edition of the Catalogue of Palaearctic Coleoptera (Pollock and Iwan 2020). In the country, the species has been found under the bark of conifers *Picea abies*, *Pinus peuce* and *Pinus* sp. at different altitudes (Kovács et al. 2011). We found this species in a spruce forest in high-mountain zone and in planted pine forest in the low-mountain zone of Vitosha Mountain, in association with *P. abies* and *P. sylvestris*, respectively. In Bulgaria, *P. depressus* appears to occur over a wide altitudinal range in the high mountains, in association with both natural and planted conifers.

*Salpingus planirostris* is widespread in Europe and also known from the Caucasus and North Africa (Morocco and Tunisia) (Pollock et al. 2020). The species was first reported from Bulgaria by Guéorguiev and Bekchiev (2009) from Western Rhodope Mountains. In the present study, we found the species in Central Stara Planina Mts as well.

*Salpingus ruficollis* is a European species, widespread on the continent (Pollock et al. 2020). Recently reported from Greece (Kakiopoulos and Demetriou 2022) and from the South European Territory of Russia (Volodchenko and Sazhnev 2023). The species has been previously reported for Bulgaria only by Angelov (1968b), from Western Stara Planina and this report was later cited by Guéorguiev and Bekchiev (2009). However, *S. ruficollis* is not included for Bulgaria in the second edition of the Catalogue of Palaearctic Coleoptera (Pollock et al. 2020). Here, we provide data on the occurrence of *S. ruficollis* in Rila and Sarnena Sredna Gora Mountains and confirm the presence of this species in Bulgaria.

*Vincenzellus ruficollis* is fairly widespread in Europe (reaching Sweden in the north) (Pollock et al. 2020). It has recently been reported from Greece (Kakiopoulos and Demetriou 2022) and Iran (Mazandaran Province) (Farashiani et al. 2022). According to Nikitsky (2016), the species occurs also in Russia (Central and South European Territory), which is not recorded in the second edition of the Catalogue of Palaearctic Coleoptera (Pollock et al. 2020). This species was reported as new to Bulgaria by Guéorguiev and Bekchiev (2009) from Belasitsa Mountain. However, in fact, the first available report of the species for the country is by Horion (1956), from Krichim (south-central Bulgaria). Here, we provide data on the occurrence of *V. ruficollis* in Central Stara Planina Mountain.

The available literature data on the distribution of the species of the families Mycteridae, Pythidae and Salpingidae in Bulgaria are scarce. Original records can be found in only twelve publications: Joakimov (1904), Nedelkov (1909), Roubal (1931), Roubal (1932), Panin (1941), Horion (1956), Angelov (1968a), Angelov (1968b), Guéorguiev and Bekchiev (2009), Guéorguiev and Ljubomirov (2009), Kovács et al. (2011) and Gradinarov and Petrova (2024). The species of these families do not appear to be rare in Bulgaria, but rather are neglected by collectors or the data on their finding are not usually published. Moreover, older publications are often unavailable and unknown to most researchers. Thus, three of the focal species in the present study (*M. tibialis*, *P. depressus* and *S. ruficollis*) were omitted from the second edition of the Catalogue of Palaearctic Coleoptera for Bulgaria and three have been reported as new to the country (*M. tibialis*, *P. depressus* and *V. ruficollis*) despite the presence of earlier records in literature from the 20^th^ century.

## Supplementary Material

XML Treatment for Mycterus (Eutryptes) tibialis

XML Treatment for Mycterus (Mycterus) curculioides

XML Treatment for Pytho depressus

XML Treatment for Salpingus planirostris

XML Treatment for Salpingus ruficollis

XML Treatment for Vincenzellus ruficollis

XML Treatment for Coleoptera

XML Treatment for Polyphaga

XML Treatment for Tenebrionoidea

XML Treatment for Mycteridae

XML Treatment for Mycterinae

XML Treatment for Mycterus

XML Treatment for Eutryptes

XML Treatment for Mycterus (Eutryptes) tibialis

XML Treatment for Mycterus

XML Treatment for Mycterus (Mycterus) curculioides

XML Treatment for Pythidae

XML Treatment for Pytho

XML Treatment for Pytho depressus

XML Treatment for Salpingidae

XML Treatment for Salpinginae

XML Treatment for Lissodema

XML Treatment for Lissodema denticollis

XML Treatment for Salpingus

XML Treatment for Salpingus planirostris

XML Treatment for Salpingus ruficollis

XML Treatment for Vincenzellus

XML Treatment for Vincenzellus ruficollis
